# Beyond safety: adverse events and unanticipated advantages of SGLT2 inhibitors

**DOI:** 10.1007/s00228-026-04023-9

**Published:** 2026-03-11

**Authors:** Lorenzo Falsetti, Nicola Tarquinio, Luciano Mucci, Silvia Santini, Emanuele Guerrieri, Laura Giovenali, Giulia Pierdomenico, Vincenzo Zaccone, Giovanna Viticchi, Gianluca Moroncini

**Affiliations:** 1 Dipartimento di Scienze Cliniche e Molecolari, Università Politecnica delle Marche, Ancona, Italy; 2 Dipartimento Percorsi Medici, U.O.C. Medicina Interna, INRCA-IRCCS, Presidio Ospedaliero di Osimo, Ancona, Italy; 3 UOC Medicina Interna, Ospedale Santa Maria della Misericordia Urbino, Azienda Sanitaria Locale 1, Pesaro-Urbino, Italy; 4 Scuola di Specializzazione in Medicina d’Emergenza-Urgenza, Università Politecnica delle Marche, Ancona, Italy; 5 Pronto Soccorso, Dipartimento di Emergenza-Urgenza, Azienda Ospedaliero-Universitaria Delle Marche, Ancona, Italy; 6 Dipartimento di Emergenza-Urgenza, Medicina Interna Generale e Subintensiva, Azienda Ospedaliero-Universitaria delle Marche, Ancona, Italy; 7 Dipartimento di Medicina Sperimentale e Clinica, Università Politecnica delle Marche, Ancona, Italy

**Keywords:** SGLT2 inhibitors, Adverse events, Genitourinary infections, Ketoacidosis, Acute kidney injury, Fractures, Amputation, Erythrocytosis, Heart failure, Chronic kidney disease

## Abstract

**Background:**

Sodium-glucose transporter 2 inhibitors (SGLT2i) are widely used for diabetes management and have demonstrated benefits in treating acute and chronic heart failure (HF) and chronic kidney disease (CKD). Their increasing application has revealed various side effects, although only a few are currently recognized as drug-related adverse events, based on ongoing observations.

**Aims:**

This review synthesizes positive and negative side effects linked to SGLT2i and proposes management strategies.

**Methods:**

A literature search of PubMed/EMBASE, Web of Science, and Google Scholar from the past 10 years identified randomized trials, meta-analyses, observational cohorts, and pharmacovigilance studies reporting SGLT2i-related events across genitourinary, endocrine, metabolic, hematologic, skeletal, and vascular domains.

**Results:**

Data indicate an increased incidence of genital infections (GIs) in diabetic subjects, while associations with urinary tract infections (UTIs) are less consistent. Non-diabetic HF/CKD patients show modest increases in GIs and UTIs. SGLT2i modestly increase hematocrit and may reveal clonal erythrocytosis. Rare conditions like Fournier’s gangrene have been reported, though without a clearly increased risk in clinical trials: all the reported cases led to drug discontinuation. There is no conclusive evidence linking SGLT2i to fractures or osteoporosis, and risk of lower-limb amputation appears comparable to DPP-4 inhibitors, with some trend compared to GLP-1 receptor agonists, suggesting caution in patients with peripheral artery disease. Observational data suggest SGLT2i may protect against syncope and reduce acute kidney injury.

**Conclusions:**

Overall, SGLT2i are safe, with manageable adverse events such as GIs and UTIs. A thorough understanding of potential complications is essential for clinicians to optimize patients’ management.

**Graphical Abstract:**

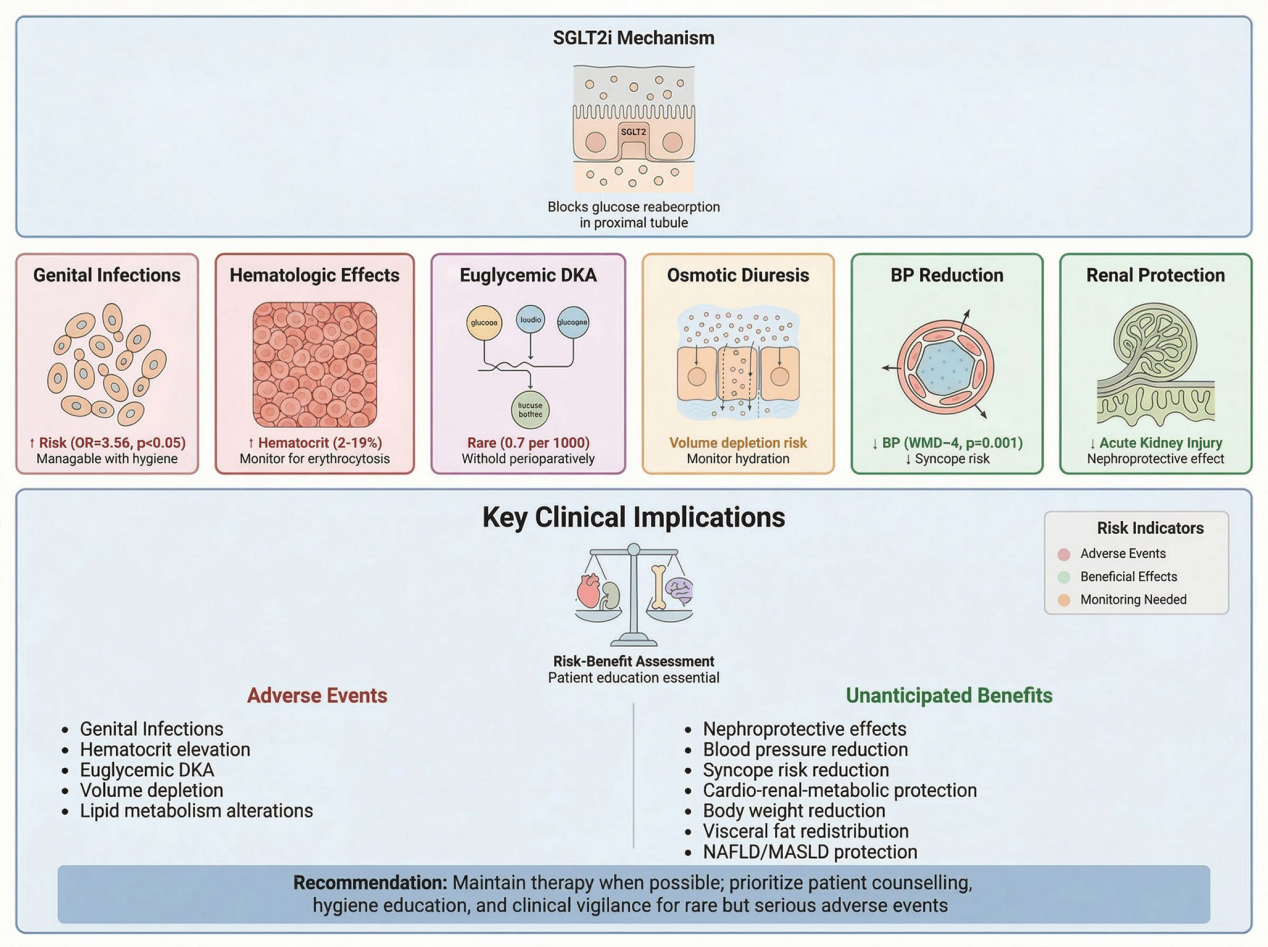

**Supplementary Information:**

The online version contains supplementary material available at 10.1007/s00228-026-04023-9.

## Introduction

Sodium–glucose cotransporter 2 inhibitors (SGLT2i) constitute a pharmacological class with a shared, well-defined mechanism of action: inhibition of renal SGLT2 in the proximal tubule, leading to reduced glucose reabsorption and increased urinary glucose excretion, accompanied by natriuresis. Although individual agents differ in pharmacokinetic properties such as SGLT2 selectivity, bioavailability, half-life, and metabolism, these differences do not alter the fundamental renal mechanism underlying their clinical effects. Four distinct molecules are currently approved both by the Food and Drug Administration (FDA) and European Medicine Agency (EMA), namely canagliflozin, dapagliflozin, empagliflozin, and ertugliflozin. Two other drugs, bexagliflozin and sotagliflozin, are currently FDA-approved but not marketed in the European Union. These molecules will not be included in this review due to their limited availability and because of the different mechanisms of sotagliflozin, an SGLT1/SGLT2 inhibitor that regulates glucose metabolism in the kidney and gastrointestinal tract. SGLT2i transitioned from glucose-lowering agents to foundational cardio–renal–metabolic treatments, with proven benefits in heart failure (HF) and chronic kidney disease (CKD). The first studies were specific for subjects with type 2 diabetes mellitus (T2DM) [1–5], while later trials observed the same beneficial effects both in diabetic and non-diabetic subjects [6, 7]. In the setting of T2DM, SGLT2i have been shown to confer significant clinical benefits compared to standard care, such as a reduction in all-cause mortality, incidence of major adverse cardiovascular events, hospitalisations due to HF, CKD progression, the occurrence of severe hypoglycemia, and significant weight loss, as shown in Table 1 [8]. In T2DM, a triple antidiabetic drug regimen including SGLT2i significantly enhances glycemic control, promotes weight reduction, and improves blood pressure regulation compared to dual therapy programs excluding SGLT2i [9]. In non-diabetic individuals, SGLT2i have demonstrated improvements in HF-related outcomes, such as decreased hospitalisations and cardiovascular mortality [10]. Following the publication of pivotal randomized clinical trials (RCTs) on dapagliflozin and empagliflozin, clinical guidelines have updated their recommendations, now endorsing these agents as first-line therapy for HF, regardless of ejection fraction [11–15]. Additionally, they positively impact CKD-related outcomes, including slowing the decline of estimated glomerular filtration rate (eGFR) and reducing albuminuria [16]. These findings allowed an expanded indication for gliflozins in this clinical setting as well [6, 7]. SGLT2i also enhance blood pressure regulation and metabolic parameters, notably by promoting weight loss, decreasing liver enzyme levels in non-alcoholic fatty liver disease, and contributing to favorable composite cardiorenal outcomes [17–19]. Thus, SGLT2i utilisation increased and is projected to grow further in the following years [20]. Clinical studies have documented a wide range of beneficial and adverse effects, which are expected to rise with increased SGLT2i prescription, underscoring the importance of surveillance and research in this field [21]: recent pharmacovigilance reports have documented an increased incidence of adverse events in real-world settings and highlighted a discrete variability in safety profiles among different gliflozins [22]. SGLT2i have also shown unexpected protective effects that should always be considered when considering their prescription. While numerous reviews have focused on either the efficacy or safety of SGLT2 inhibitors, fewer have integrated adverse events and pleiotropic effects within a unified clinical decision-making framework. Moreover, several effects initially perceived as safety concerns have subsequently emerged as mechanistically informative or even protective in selected populations. With this narrative review we aimed to summarise the adverse events and unanticipated advantages of SGLT2i, offering pragmatic information for routine practice.

## Search strategy and selection criteria

The study group conducted a structured search in PubMed/EMBASE, Web of Science, and Google Scholar. The research was limited to systematic reviews, meta-analyses, observational studies and case series and case reports published in the last 10 years, in English language. The recent approval of dapagliflozin and empagliflozin for T2DM in subjects aged 10–17 years as add-ons to diet and exercise represents a clinically significant and evolving area [23]. However, in this review, we focused on adult patients, including only studies that enrolled subjects aged 18 years or older. The study group selected SGLT2i-related adverse events and the unanticipated advantages of these drugs by analysing the most recent literature, particularly pharmacovigilance studies [21–28]. The group of reviewers prepared a list of SGLT2-related events and searched the MeSH database for Major topics, compiling a list of strings for literature research. The MeSH strings adopted for PubMed/EMBASE search in each paragraph are synthesised in the Supplementary Materials. The list of retrieved papers was analysed by the study group, that selected by consensus the most influential papers for each topic. The group prioritized systematic reviews and meta-analyses, as well as RCTs and observational cohort studies, addressing SGLT2i-related safety outcomes, organizing the results by organ systems. When evidence was conflicting, we emphasised the most recent meta-analyses and reported the uncertainty.

## Narrative review

Most adverse and pleiotropic effects of SGLT2 inhibitors derive from three interconnected mechanisms: glycosuria-induced metabolic shifts, natriuresis-mediated hemodynamic changes, and modulation of intrarenal oxygen handling. Pharmacovigilance data indicate that the most frequently reported events associated with SGLT2i use include endocrine disturbances (diabetic ketoacidosis, euglycemic diabetic ketoacidosis, and bone fractures), vascular diseases (syncope, amputations, hypotension, and dehydration), renal and urogenital diseases (Fournier gangrene, genital and urinary tract infections, nephrolithiasis, and acute kidney injury), hematologic disorders (erythrocytosis) and metabolic effects (lipid metabolism alterations, body weight reduction, non-alcoholic fatty liver disease reduction) [19, 21, 25–27, 29–31]. The most recent meta-analytic evidence indicates that SGLT2i significantly increase the risk of genital mycotic infections (GIs) and euglycemic diabetic ketoacidosis (EDKA), especially in T2DM [32, 33]. Meta-analyses further reveal alterations in lipid metabolism, with SGLT2 inhibitors modestly increasing HDL-C and LDL-C while reducing triglycerides relative to controls across large RCT populations. There is uncertainty regarding the association between SGLT2i use and several reported adverse events: recent evidence indicates that the incidence rates of hypovolemia, acute kidney injury (AKI), urinary tract infections (UTIs), diabetic ketoacidosis (DKA), fractures, amputations, and severe hypoglycemia do not differ significantly from those observed with placebo [34]. SGLT2 inhibition consistently produces modest but statistically significant reductions in body weight and decreases in BMI versus controls, reflecting caloric loss via glucosuria and attendant fat mass reduction [31]. Imaging-based RCTs and meta-analyses in patients with NAFLD/MASLD show significant reductions in hepatic fat content and improvements in liver enzymes and proton density fat fraction compared with placebo or standard care, indicating potential benefits on ectopic hepatic lipid accumulation [16]. Table 1 provides a summary of the known side effects, the direction of the risk, the key studies considered, and current recommendations for clinical management.

**Table 1:** synthesis of SGLT2i-related events, risk direction, key studies and management suggestions

AE	Direction	Prevalence and Risk	Key Studies	Suggestions
**GIs**	Increased risk	*Prevalence* - Males: 2–5%; Females: 10–15% *Risk* - RR: 3.56; 95%CI: 2.84–4.46	Lin 2021 [35]; Marilly 2022 [36]; Shi 2023 [32]; Sridharan 2024 [37];	Hygiene counselling; maintain therapy in mild to moderate cases; discontinue only in severe or life-threatening infections.
**UTIs**	Neutral	*Prevalence* − 7% (versus 6.5% of placebo) *Risk* - RR: 1.06; 95%CI: 1.00-1.12	Li 2017 [38]; Puckrin 2018 [39]; Dave 2019 [40]; Kittipibul 2024 [41];	Do not discontinue for uncomplicated UTIs; monitor in recurrent or anatomically complicated cases.
**FG**	Neutral Rare event	*Incidence* - Rare, < 1/10.000 *Risk* - No increased risk in RCTs	FDA 2018 [42]; Silverii 2020 [43]; Liu 2024 [44]; Azmi 2025 [45];	No routine screening: discontinue SGLT2i permanently if the event occurs.
**AKI**	Decreased Risk	*Prevalence* − 2.18% *Incidence* - HR: 0.64; 95%CI: 0.59–0.70	Menne 2019[46]; Ma 2023[47]; Wang 2024 [48];	Nephroprotective effect; monitor eGFR in frail, elderly patients; caution is advised with dehydration or diuretics.
**Nephrolithyasis**	Decreased Risk	Nephrolythiasis *versus* placebo - OR: 0.61; 95%CI: 0.53–0.70 Nephrolythiasis *versus* GLP-1 or Nephrolythiasis *versus* DPP-4i - OR: 0.66; 95%CI: 0.47–0.93	Kanbay 2025 [49]; Balasubramanian 2022 [50];	Reduced risk of kidney stones, possibly via increased urinary flow and reduced uric acid supersaturation.
**Erythrocytosis**	Increased Risk	*Prevalence* − 2–19% of patients *Effect* - mean Hb increase: 2–2.5 g/dL - mean Hct increase: 2–4%	Gangat 2023 [51]; Chen 2024 [29];	Exclude alternative causes; consider discontinuation if Hct is greater than 53%.
**EDKA**	Increased Risk	*Incidence* − 0.7–1.6/1000 patient/year *Risk* - OR: 2.13 (95% CI: 1.38–3.27)	Peters 2015 [52]; Menne 2019[46]; Shi 2023 [32]; ADA Consensus 2023 [53];	Prevention: Educate patients; avoid excessive insulin dose reduction; discontinue 3 days before surgery; maintain hydration and carbohydrate intake. Treatment: early recognition is very important; manage as DKA
**Fractures** **BMD Reduction**	Neutral	*Prevalence* − 1.0-1.6% *Risk* Bone fractures - OR: 0.86; 95%CI: 0.70–1.06 Bone fractures *versus* DPP4i - HR:0.90; 95%CI:0.73–1.11 Bone fractures *versus* GLP1RA - HR: 1.00; 95%CI: 0.80–1.25 Bone Mineal Density: - Lumbar spine: WMD: -0.02; 95%CI:-0.38-0.34; - Femoral neck: WMD: 0.11; 95%CI:-0.28-0.50; - Total hip: WMD: -0.20, 95% CI: -0.41-0.01; - Distal forearm: WMD: -0.20; 95%CI: -0.62-0.22;	Neal 2017 [4]; Cheng 2019 [54]; Zhuo 2021 [55]; Wang 2023 [56];	Assess baseline osteoporosis risk and follow standard guidelines for osteoporosis and fall prevention.
**Hypotension** **Hypovolemia**	Mild Risk Increase	*Prevalence* − 1.2–3.4% *Effect* - Mean SBP reduction of 4 mmHg *Risk* Hypovolemia in overall subjects - Not increased risk Hypovolemia in heart disease - RR: 1.21; 95%CI:1.06–1.39 Hypotension (asymptomatic) - OR: 1.17; 95%CI: 1.03–1.33 Hypotension (symptomatic) - Not increased risk Orthostatic hypotension in T2DM - RR:1.17; 95%CI:0.65–2.09	McGuire 2020 [57]; Younes 2022 [58]; Cao 2022 [59]; Goldman 2023 [26]; Mitsuboshi 2025 [60];	Monitor hydration status, especially in the elderly, those with heart failure, and polytherapy; adjust diuretics as needed.
**Syncope**	Decreased Risk	*Prevalence* − 0.5–1.2% *Risk* New-onset syncope *versus* DPP4i - HR: 0.49; 95%CI: 0.41–0.57 Vasovagal syncope - HR: 0.55; 95%CI: 0.32–0.93 - Modest increase in frail, elderly patients with volume depletion	Gao 2024 [61] Sardu 2022 [62]	In T2DM with recurrent vasovagal syncope SGLT2i have a potential benefit. In elderly/frail patients: monitor orthostatic blood pressure, review antihypertensive drugs, and ensure adequate hydration. Adjust diuretics as needed and avoid routine discontinuation.
**Amputations**	Neutral	*Incidence* − 1.5–5.0/1.000 patients/year in T2DM *Risk* Amputations versus placebo - HR:0.98; 95%CI:0.68–1.41 Amputations versus DPP4i - HR: 0.98; 95%CI:0.73–1.31 Amputations versus GLP1RA - HR:1.26; 95%CI:0.99–1.60	Neal 2017 [4]; Barbarawi 2022 [33]; Mizutani 2022 [63]; Lu 2023 [64];	No routine discontinuation; closely monitor high-risk subgroups (PAD, prior ulcers/amputations, women, high-dose statins).
**Body ** **weight reduction ** **Viseral fat redistribution**	Decreased Risk	*Effect* - Body weight reduction by 1.5–2.5 kg *versus* placebo over 24–52 weeks; primarily fat mass loss;	Cheong 2022 [31] Wang 2023 [65]	BMI reduction: beneficial metabolic effect; Visceral fat redistribution: cardiometabolic risk reduction. Consistent effects across T2DM and non-diabetic populations
**Lipid metabolism alterations**	Increased Risk	*Effect* - Modest increase in LDL-C (2–5%), and in HDL-C (3–8%), reduction in triglycerides (5–10%) - The blood lipid alteration is overall neutral to favorable atherogenic profile	Bechmann 2023 [30]	Monitor lipid profile after initiation; LDL rise is usually small and offset by cardiovascular and cardiometabolic benefits
**NAFLD** **MASLD**	Decreased Risk	*Effect* - Reduction of liver fat content; significant reduction of liver enzymes; modest improvement in fibrosis markers - Liver effects are independent of weight loss	Kuchay 2018 [66] Dwinata 2020 [67] Lopez 2024 [68] Abbas 2025 [19]	Potential disease-modifying benefit; consider in T2DM with NAFLD; not yet approved as NAFLD therapy
**All-cause mortality**	Decreased Risk	*Risk* All-cause mortality - HR: 0.88; 95%CI: 0.82–0.94 Cardiovascular death - HR: 0.86; 95% CI: 0.81–0.92 Heart Failure death - HR: 0.68; 95%CI: 0.46–1.02 Sudden Cardiac death - HR: 0.86; 95% CI: 0.78–0.95 Non-cardiovascular death - HR: 0.91; 95% CI: 0.79–1.05	Patel 2024 [69]	Multisystem effects, SGLT2i use reinforces the net clinical benefit

*AE adverse event, AKI, acute kidney injury, BMI body mass index, BMD bone mineral density, DDP4i dipeptidyl peptidase-4 inhibitors, EDKA euglycemic diabetic ketoacidosis, eGFR estimated glomerular filtration rate, FG Fournier’s Gangrene, GI genital mycotic infection, GLP1RA glucagon-like peptide-1 receptor agonist, Hb haemoglobin, Hct hematocrit, HR hazard ratio, MASLD Metabolic dysfunction-Associated Steatotic Liver Disease, NAFLD Non-alcoholic fatty liver disease, PAD peripheral artery disease, RR rate ratio, SBP systolic blood pressure, SGLT2i sodium glucose co-transporter 2 inhibitors, T2DM type 2 diabetes mellitus, UTIs urinary tract infections, WMD weighted mean difference*

### Renal and urogenital manifestations

#### Genitourinary Infections

Genitourinary infections are the most commonly reported adverse events associated with SGLT2i use, driven by glucosuria-induced changes in the urogenital environment and occurring predominantly as mild-to-moderate infections in susceptible individuals. RCTs and meta-analyses report a markedly increased risk of genital infections and a small, non-significant increase in UTIs, with a higher risk in diabetic than non-diabetic subjects [2, 4, 35, 70].

- *UTIs in diabetic subjects*: T2DM is a significant risk factor for UTIs. Affected individuals show an increased UTI prevalence with respect to non-diabetic individuals, and this effect is particularly evident among women [71, 72]. This association has been linked to several factors, particularly glycosuria, impaired immune response, diabetic autonomic neuropathy, and increased adherence to urothelial cells [71, 72]. However, the association between SGLT2i and an increased UTIs risk remains controversial [73]: cohort studies observed that, among diabetic subjects, UTIs occur more frequently in patients undergoing SGLT2i, and a raised risk was also observed in the FDA adverse events reporting system [74, 75]. On the other hand, several retrospective studies, meta-analyses, and RCTs did not confirm this association, underscoring the absence of an increased risk when matching the treatment arm with both a placebo and an active comparator [38, 39, 76]. Specifically, comparing SGLT2i with other second-line medications for diabetes did not show an increased risk of complicated and non-complicated UTIs in T2DM [40]. In 2015, post-marketing surveillance indicated progression to life-threatening urosepsis or pyelonephritis in individuals with T2DM treated with canagliflozin, prompting the FDA to issue a warning regarding the potential risk of serious UTIs in patients receiving SGLT2i [41]. Nevertheless, more recent reports have emphasised that SGLT2i do not seem to raise the risk of severe infections, such as urosepsis and pyelonephritis [40, 71]. According to current evidence, given the cardiovascular benefits of the treatment, SGLT2i should not be discontinued solely due to the occurrence of uncomplicated UTIs, unless there are anatomical or functional alterations of the urinary tract or a history of recurrent UTI [77].
- *UTIs in non-diabetic subjects*: individuals without T2DM have a significantly lower risk of developing UTIs due to the absence of diabetes. SGLT2i are likely associated with an increase in benign urinary symptoms attributable to osmotic diuresis, which may potentially result in a higher diagnostic rate of infections among patients prescribed these medications [78]. Recent meta-analyses have highlighted that, despite a smaller number of studies, non-diabetic individuals treated with SGLT2i have a slightly higher UTIs risk compared to untreated individuals, who, in turn, have a lower risk than T2DM [79, 80]. Considering specific diseases, meta-analyses did not find a significantly increased risk in CKD, and a marginal increase in HF [58, 59, 81].
- *GIs in diabetic subjects*: most published studies agree on a heightened GIs risk, especially of mycotic origin, linked to SGLT2i treatment compared to other antidiabetic regimens [37, 60, 82–87]. A recent meta-analysis considering subjects with T2DM and a high CV risk observed a clear benefit in terms of reduction of all-cause death, major adverse cardiovascular events, HF and end-stage renal disease, counterbalanced by an increased risk of GIs and diabetic ketoacidosis [36]. DPP-4 inhibitors (DPP-4i) could be considered as an option for individuals intolerant to SGLT2i due to GIs [88, 89].
- *GIs in non-diabetic subjects*: trials and meta-analyses have shown that HF and CKD are associated with an increased GIs risk during SGLT2i treatment: both HF and CKD subjects face a 3-fold increase in GIs risk when treated with SGLT2i [58, 81]. Over time, clinical practice considerations have emerged regarding SGLT2i-associated GIs, mainly focusing on patients with cardiovascular disease [41].
- *Fournier’s gangrene*: Fournier’s gangrene (FG) is a severe infection affecting the soft tissues, the perineum fascia and the genital region associated with high morbidity and mortality rates. Preliminary observations linked FG to SGLT2i use, prompting the FDA and other regulatory agencies to caution against this potential side effect [42, 90]. A recent review of published cases confirmed that FG is uncommon, even during SGLT2i treatment [44]. Nevertheless, despite the limited number of observed events, a comprehensive meta-analysis did not identify an increased FG risk in diabetic individuals undergoing SGLT2i treatment compared to those receiving a placebo or alternative antidiabetic therapies [43]. FG remains a rare but potentially life-threatening complication that should prompt the immediate SGLT2i discontinuation [45].
- *Management of genitourinary complications*: UTIs and GIs management in patients treated with SGLT2i should follow a structured approach based on four complementary principles [41]. First, all patients should undergo a careful assessment of risk factors before initiating therapy. Particular attention should be paid to glycemic control, local anatomical abnormalities such as obstructive uropathy, and a history of recurrent UTIs or GIs, to identify individuals requiring closer monitoring. Second, universal counselling plays a key role in prevention: patients should be educated to recognise early signs and symptoms of infection and to adopt adequate hygiene practices [91, 92]. Preventive measures include maintaining proper hydration, avoiding urine retention, urinating before sexual activity, limiting the use of irritant intimate products, and wearing breathable cotton underwear. In women, additional attention to menstrual hygiene, with regular changing of sanitary pads or tampons, as well as daily cleansing and drying of the genital area, may further reduce the risk of GIs [77]. Third, infections that develop during therapy should be managed according to their severity. Most cases are mild to moderate and can be treated with standard oral antibiotics for UTIs or topical or systemic antifungals for GIs, without discontinuing SGLT2i therapy [77]. Even in severe but clinically stable cases, continuation of the drug can often be considered, given its established cardiorenal benefits. Interruption should remain limited to unstable or life-threatening conditions, such as urosepsis, pyelonephritis, or FG, where immediate substitution with an alternative glucose-lowering agent is recommended [45]. Finally, once an acute severe infection has been resolved and the patient is hemodynamically stable, reinitiation of SGLT2i therapy is generally appropriate. If the drug was discontinued for a non-severe infection, reintroduction should be considered without delay once symptoms have subsided and no contraindications are present. This pragmatic strategy balances infection management with the need to preserve the long-term cardiovascular and renal protection conferred by this class of drugs [93].

#### Acute kidney injury 

Acute kidney injury associated with SGLT2i therapy is uncommon and largely transient, typically related to early hemodynamic changes rather than intrinsic nephrotoxicity, with overall long-term renoprotective effects predominating. A transient decline in eGFR is commonly observed within the first weeks of SGLT2i therapy, reflecting hemodynamic changes due to reduced intraglomerular pressure. This “early dip” is usually modest, clinically insignificant, and followed by long-term stabilisation and a slower decline in renal function, as observed with renin–angiotensin system inhibitors. Current guidelines emphasise that this phenomenon should not prompt treatment discontinuation unless the reduction exceeds 30% from baseline or occurs in the setting of an acute illness [94–97]. In fact, most evidence indicates the nephroprotective effects of SGLT2i, particularly in reducing the risk of AKI. Compared with placebo, SGLT2i significantly lowers the risk of composite renal outcomes. This benefit was observed across all baseline eGFR categories, including patients with CKD [47]. This reduction is also supported by large meta-analyses of published trials and seems uniform in all the renal function strata [46, 98, 99].The benefits of SGLT2i in different AKI subgroups are shown in Table 2.

- *AKI in subjects with normal renal function*: In patients with preserved renal function, the occurrence of severe AKI is very low (< 1%). In this subgroup, SGLT2i are safe, improve renal function, and reduce the risk of severe AKI. Age ≥ 65 years remains the strongest AKI predictor in this population [98].
- *AKI in subjects with chronic kidney disease*: In this setting, the absolute AKI risk is higher, yet SGLT2i maintain consistent renoprotective effects. In individuals with an eGFR < 60 mL/min/1.73 m², SGLT2i reduced the risk of primary renal outcomes. The benefit is similar across CKD subgroups: 38% reduction for eGFR 45–60 mL/min/1.73 m², 29% for eGFR 30–45 mL/min/1.73 m², and 32% for eGFR < 30 mL/min/1.73 m². Of note, SGLT2i also reduced secondary outcomes - including sustained decline in kidney function, progression to end-stage kidney disease, and renal or cardiovascular death - by approximately 33% in patients with reduced eGFR [99, 100]. Although data remain limited in patients with eGFR < 30 mL/min/1.73 m², available meta-analyses suggest that SGLT2i may still provide renal protection in this subgroup, warranting further dedicated trials [99, 100].
- *AKI in other diseases*: Beyond CKD, SGLT2i are associated with reduced AKI risk in other populations. In T2DM, treatment lowered AKI risk [101]. In HF, SGLT2i reduced the risk of AKI progression, as shown in Table 2 [48].
- *Management of acute kidney injury*: No significant increase in renal adverse events has been reported with SGLT2i. Overall, SGLT2i appear safe and effective in most patients, including those with impaired kidney function. Nevertheless, careful monitoring is recommended in individuals at higher risk, such as the elderly or those with pre-existing CKD. Current guidelines emphasise baseline eGFR assessment before therapy initiation and close monitoring during intercurrent illnesses or acute kidney impairment. In these circumstances, temporary discontinuation may be considered, particularly in situations predisposing to hypovolemia or reduced renal perfusion, such as severe infections, dehydration, surgery, or exposure to nephrotoxic agents. At the same time, therapy should be reinitiated promptly once the patient is stable. This “sick day management” strategy, endorsed by KDIGO and ADA, allows clinicians to maximise the long-term cardiorenal benefits of SGLT2i while minimising AKI risk in vulnerable patients [97, 102].

A synthesis of the AKI risk reduction effects by SGLT2i in different patients’ populations is shown in Table 2.

**Table 2:** Acute kidney injury risk with SGLT2i in different populations

Population	Effect	Direction	Key studies	Interpretation
**T2DM**	*AKI Incidence* − 11.5 per 1000 patients/year *Risk* - RR: 0.82 (95%CI: 0.73–0.92)	Decrease 25–35%	Zinman 2015 [70] Neal 2017 [4] Wiviott 2018 [2]	Consistent AKI risk reduction across RCTs
**CKD**	*AKI Prevalence* − 2.18% (SGLT2i) *versus* 2.55% (placebo) *Risk* *Composite renal events*: - HR: 0.64 (95%CI:0.59–0.70) *Composite renal events*,* eGFR < 60 ml/min/m*^*2*^ - HR: 0.70 (95%CI:0.58–0.83) *Composite renal events*,* eGFR 45–60 ml/min/m*^*2*^ - HR: 0.62 (95%CI:0.47–0.82) *Composite renal events*,* eGFR < 30 ml/min/m*^*2*^ - HR: 0.71 (95%CI:0.57–0.87)	Decrease 25–40%	Neuen 2019 [100] Heerspink 2020 [6] Herrington 2022 [7] Neuen 2025 [103]	Renal protective effect despite initial eGFR dip
**HF**	*AKI Prevalence* − 1.8% (SGLT2i) *versus* 2.4% (no SGLT2i) *Risk* - RR: 0.72 (95%CI: 0.61–0.85)	Decrease 20–30%	McMurray 2019 [11] Packer 2020 [13] Anker 2021 [12]	
**Overall**	*AKI Prevalence* - Rare, < 1% *AKI risk in subjects with normal renal function* - OR: 0.64 (95%CI: 0.53–0.78)	Decrease 23–36%	Menne 2019 [46] Li 2021 [99] Ma 2023 [16] Ziser 2024 [98]	Class effect is supported by pooled evidence.

*AKI acute kidney injury, CKD chronic kidney disease, eGFR estimated glomerular filtation rate, HF heart failure, HR hazard ratio, RR risk ratio, OR odds ratio, T2DM type 2 diabetes mellitus, RCT randomized controlled trial*

#### Nephrolithiasis 

SGLT2i appear to reduce the risk of nephrolithiasis, potentially through favorable effects on urinary composition, including increased urine volume and reduced supersaturation of lithogenic salts. Nephrolithiasis is a prevalent condition worldwide, with recurrence rates following the initial event reaching up to 50%. The pathophysiology involves supersaturation of urine with lithogenic substances, decreased urinary volume, and low urinary pH [104]. T2DM is a known risk factor for nephrolithiasis and may exacerbate this condition through mechanisms such as hyperuricemia and increased uricosuria. Recent studies suggest that SGLT2i use is associated with a 39% lower risk of nephrolithiasis compared to placebo or active controls, as shown in Table 1 [49]. This protective effect is hypothesised to result from increased urinary flow due to osmotic diuresis, leading to dilution of lithogenic substances. Moreover, SGLT2i have been associated with increased urinary citrate excretion, a known inhibitor of calcium salt crystallisation [50]: animal studies have demonstrated increased urinary bicarbonate excretion and higher urine pH following SGLT2i, which may further reduce the risk of stone formation, and observational studies underlined a beneficial effect in the urine metabolome in a glucose-lowering independent way [105, 106]. Further RCTs are necessary to confirm the efficacy of SGLT2i in this setting, particularly in individuals without diabetes. Until such data are available, SGLT2i in nephrolithiasis management should be considered only in patients with other established indications.

### Hematologic adverse events

#### Polycythemia

SGLT2i–Erythrocytosis associated erythrocytosis reflects stimulation of erythropoiesis through improved renal oxygenation and altered iron metabolism and may unmask previously unrecognized hematologic conditions in predisposed individuals. All four SGLT2i have been shown to modestly increase hematocrit levels by approximately 2–4% compared with placebo [107]. More marked erythrocytosis has been reported in 2–19% of treated patients [108], typically emerging after a median of nine months of therapy, with an average haemoglobin increase of 2.5 g/dl and a hematocrit rise of about 7.5%, as shown in Table 1 [109]. Several mechanisms have been proposed to explain this phenomenon. First, osmotic diuresis may cause hemoconcentration, reflected by higher hematocrit in the absence of increased erythrocyte or reticulocyte counts. Second, SGLT2i modulate iron metabolism, with reductions in hepcidin and ferritin levels accompanied by enhanced erythropoietin production [110]. Third, a decline in renal parenchymal oxygenation at the corticomedullary junction may activate hypoxia-inducible factor (HIF)-2α while inhibiting HIF-1α, thereby stimulating erythropoietin release from peritubular interstitial cells [111, 112]. Consistently, several case reports and series have documented erythrocytosis in patients receiving SGLT2i [109, 113, 114]. Importantly, erythrocytosis should be considered secondary to SGLT2i use only after excluding more common etiologies. Since the earliest reports, it has been recognised that SGLT2i may unmask pre-existing clonal disorders, such as polycythemia vera (PV), or exacerbate other forms of secondary erythrocytosis, including that induced by testosterone therapy [115–117]. Current thresholds defining erythrocytosis, hemoglobin > 16.5 g/dL or haematocrit > 49% in men, and haemoglobin > 16 g/dL or haematocrit > 48% in women, should prompt a full diagnostic work-up [51]. In patients on SGLT2i, levels above these cut-offs have been reported, including cases with severe erythrocytosis and hematocrit exceeding 53% [118]. Physicians should conduct a comprehensive evaluation to identify alternative or concomitant causes, including detailed medical history, physical examination, complete blood count with reticulocyte analysis, iron status assessment (iron, ferritin, transferrin), erythropoietin levels, and molecular testing for JAK2 mutations. The priority must be to exclude PV, which requires specific management, but also assess other causes of secondary erythrocytosis [119, 120]. SGLT2i treatment can reveal previously undiagnosed PV, and drug discontinuation in such cases has been associated with a marked improvement in hematological parameters [115].

### Endocrine adverse events

#### Euglycemic diabetic ketoacidosis

Euglycemic diabetic ketoacidosis is a rare but potentially serious complication of SGLT2i therapy, arising from altered insulin–glucagon balance and enhanced ketogenesis under specific precipitating conditions. The incidence of diabetic ketoacidosis (DKA) associated with SGLT2i in T2DM ranges from 0.7 to 1.6 per 1.000 patient-years, with overall mortality reported up to 5% in experienced centres [121, 122]. Notably, between one-third and two-thirds of SGLT2i-treated subjects who develop DKA are euglycemic (EDKA) [123]. Two complementary pathophysiological pathways have been implicated in EDKA during SGLT2 inhibition.

- *Systemic metabolism alterations*: By increasing glycosuria and lowering plasma glucose, SGLT2i reduce circulating insulin and increase glucagon secretion (also via paracrine mechanisms), which shifts substrate utilisation toward lipolysis. The rise in free fatty acids stimulates hepatic β-oxidation and ketogenesis, increasing circulating ketone bodies [124, 125].
- *ATP turnover alterations in the proximal tubule*: Reduced sodium reabsorption with SGLT2i lowers ATP consumption in proximal tubular cells (via decreased Na⁺/K⁺-ATPase activity), which can diminish renal ammoniagenesis and the intrarenal oxidation/handling of filtered ketone bodies. In settings of heightened ketone production, these changes may contribute to bicarbonate loss and high-anion-gap metabolic acidosis.

These mechanisms create a vulnerable metabolic milieu in which specific triggers can precipitate EDKA. Four primary triggers that further reduce the insulin-to-glucagon ratio in SGLT2i users have been described [126]: (i) pre-existing insulin deficiency or excessive insulin dose reduction when initiating SGLT2i, (ii) intentional or unintentional fasting and ketogenic diets, and (iii) acute medical or surgical illness with reduced intake, including alcohol abuse. Significant insulin deficiency may be suspected from prior DKA episodes, alcohol abuse, current insulin therapy, and markedly elevated glycated haemoglobin (e.g., > 10%). Because SGLT2i increase circulating ketones, conditions that increase ketone production should be identified and mitigated - particularly prolonged fasting, ketogenic diets, anorexia, alcohol intoxication, acute gastrointestinal disorders, and gastroparesis. Pre-existing metabolic acidosis should be carefully assessed by reviewing the history of AKI or CKD and checking serum creatinine and bicarbonate. Very low serum creatinine in low-muscle-mass individuals may signal reduced capacity to metabolise ketones, suggesting a greater susceptibility [127]. Risk-reduction strategies include avoiding more than a 20% reduction in insulin dose, checking urinary ketones after insulin adjustments, maintaining adequate hydration, limiting or avoiding alcohol, and ensuring that each meal and snack contains approximately 30–35% carbohydrates. Ketogenic diets should be discouraged. For perioperative care or other situations involving prolonged fasting, SGLT2i should be withheld for three days before elective procedures. While this practice reduces EDKA risk, it may be accompanied by suboptimal postoperative glycemic control and potentially higher complication rates; thus, careful glucose management is required, and further studies are warranted [128]. During acute illness with compromised intake, patients should be counselled to maintain hydration and glucose availability (small volumes of electrolyte solutions and modest carbohydrate intake every 20 min). In emergencies requiring fasting, intravenous dextrose together with subcutaneous or intravenous insulin should be administered to preserve an adequate glucose-to-insulin ratio and suppress ketogenesis [127]. If preventive measures fail, clinicians must promptly recognise EDKA. Presenting symptoms overlap with classic DKA and include vomiting, anorexia, abdominal pain, dyspnea, and tachycardia [52, 129]. Compared with hyperglycemic DKA, EDKA often evolves more insidiously and lacks osmotic symptoms (polyuria, polydipsia), which may delay diagnosis and worsen outcomes. Once EDKA is suspected, accurate history (including SGLT2i or salicylates use) and contributory conditions (acute or chronic kidney failure, sepsis, alcohol abuse) should be assessed. Confirmatory testing includes arterial blood gas (pH, anion gap, bicarbonate), followed by serum glucose and ketone levels. EDKA, first described over 50 years ago, is currently characterized by two major criteria: blood glucose < 200 mg/dL and ketonemia > 3 mmol/L; if these are met, at least one minor criterion should be present to confirm the diagnosis: arterial pH < 7.30, serum bicarbonate < 18 mEq/L, or anion gap > 10 [130, 131]. Management mirrors that of DKA and focuses on immediate discontinuation of SGLT2i, aggressive fluid resuscitation, intravenous insulin treatment, and correction of electrolyte disturbances [132]. As with DKA, EDKA is considered resolved when all the following targets are achieved: (i) the patient can eat, (ii) pH > 7.3, (iii) bicarbonate > 15 mmol/L, and (iv) ketones < 0.6 mmol/L [133].

#### *Bone mineral density and fracture risk*

Initial concerns regarding skeletal safety with SGLT2 inhibitors have not been consistently supported by later RCTs or meta-analyses, indicating no clear increase in fracture risk at the class level. Several plausible mechanisms may explain how SGLT2i influence bone and mineral metabolism. These include minor alterations in phosphate handling that could activate the FGF23-1,25-dihydroxyvitamin D-PTH axis, as well as effects related to natriuresis that may impact bone turnover. Both mechanisms are supported by experimental and physiological evidence; for instance, effects of canagliflozin on FGF23 and vitamin D metabolism have been documented [134]. Clinically, a safety signal indicating an increased risk of fractures was initially identified in the CANVAS program involving canagliflozin, where the fracture incidence was 4.0% in the active treatment arm, compared to 2.6% with placebo. This increased risk was primarily observed in older and higher-risk subgroups, thereby raising concerns that prompted further mechanistic and outcomes investigations [4, 135]. However, subsequent pooled analyses and meta-analyses have not demonstrated a consistent class-wide increase in fracture risk: network and pairwise meta-analyses of randomized trials and more recent systematic reviews and meta-analyses find no statistically significant overall association between SGLT2i use and fracture incidence, and no consistent, clinically meaningful decline in bone mineral density (BMD) across major skeletal sites (lumbar spine, femoral neck, total hip, distal forearm), as shown in Table 1 [136]. Individual studies’ results are heterogeneous: some trials examining canagliflozin have reported modest, site-specific decreases in hip BMD over extended follow-up periods, whereas other studies have observed no significant alterations in lumbar spine or femoral neck BMD. Meta-analytic estimates available to date suggest there is no definitive class effect; however, the confidence intervals are broad, and the number of high-quality, long-term BMD trials remains limited [135]. Implications for clinical practice are pragmatic: existing evidence does not support the discontinuation of SGLT2i solely due to concerns about fractures in most patient populations. However, clinicians should evaluate the baseline fracture risk in frail or elderly individuals - particularly those with a history of fragility fractures, very low baseline BMD, high fall risk, concomitant diuretic use, or advanced CKD - using validated scoring systems. When indicated, standard osteoporosis prevention measures should be implemented, including bone density testing, falls risk reduction strategies, and pharmacologic intervention when appropriate. Ongoing pharmacovigilance and extended longitudinal studies in high-risk subpopulations, such as frail elderly individuals and patients with advanced CKD, are strongly warranted [136].

### Hemodynamic-related effects

Despite their mechanisms of action, SGLT2i are associated with modest reductions in blood pressure and a low risk of hypovolemia, as shown in Table 1. Moreover, they are associated to a sympathetic tone improvement that translates clinically into a reduced syncope risk, especially in T2DM. Volume depletion and hypotension occur mainly in patients treated with diuretics or subjects with impaired autonomic or hemodynamic reserve, who deserve particular attention.

#### Hypotension

A modest reduction in blood pressure is a well-recognised SGLT2i effect. Meta-analyses confirm that SGLT2i therapy lowers systolic blood pressure by approximately 4 mmHg (weighted mean difference [WMD] − 4.0 mmHg; 95%CI:–4.4 to − 3.5) and diastolic blood pressure by 1–2 mmHg (WMD − 1.6 mmHg; 95%CI:–1.9 to − 1.3) compared with baseline [137]. Although statistically significant, these reductions are generally of limited clinical impact. The effect is observed in both normotensive and hypertensive patients, and it may be particularly useful in hypertensive individuals at high cardiovascular risk. Whether SGLT2i induce symptomatic hypotension in normotensive subjects remains uncertain. Nonetheless, some subgroups -including the elderly, patients with neurodegenerative diseases, T2DM, HF, or those already receiving multiple antihypertensive agents- should be considered at higher risk of symptomatic hypotension, syncope, and falls [58, 138, 139]. In HF, SGLT2i slightly increase the risk of hypotension, though the risk of symptomatic hypotension does not appear to be significantly elevated, as shown in Table 1 (41,114). Case reports describe episodes of dehydration and symptomatic hypotension in patients receiving both SGLT2i and intensive guideline-directed therapy, underscoring the need for careful assessment of volume status in frail HF patients [140]. Nevertheless, the overall clinical benefits of SGLT2i consistently outweigh these risks, and discontinuation is rarely required [141] In T2DM, SGLT2i are not associated with an increased incidence of orthostatic hypotension [142]. However, given that long-standing diabetes can impair autonomic function, patients with multiple antihypertensive therapies, poor glycemic control, or prolonged disease duration may be particularly vulnerable [143]. In such individuals, SGLT2i-induced volume depletion could aggravate pre-existing orthostatic symptoms. Taken together, current evidence suggests that SGLT2i rarely cause clinically significant hypotension on their own. In elderly or frail patients, and in those with HF or diabetic autonomic neuropathy, the risk of falls should be mitigated through appropriate management: ensuring adequate hydration, reviewing and adjusting concomitant antihypertensive and diuretic therapy, and maintaining SGLT2i when possible, given their favourable cardiorenal risk–benefit profile [144]. Individuals with T2DM can develop orthostatic hypotension due to autonomic dysfunction, but hypovolemia and anti-hypertensive drugs can worsen symptoms. Thus, it could be advisable to screen subjects with T2DM for orthostatic hypotension, suggest adequate oral hydration, optimise anti-hypertensive drug treatment and maintain SGLT2i for their optimal risk/benefits ratio.

#### *Syncope*

Syncope represents a significant diagnostic and therapeutic challenge, with substantial implications for healthcare costs. T2DM has been associated with recurrent vasovagal syncope (VVS), and T2DM itself is considered an independent predictor of recurrence [145]. Autonomic dysfunction in diabetes is characterised by parasympathetic overactivity, sympathetic withdrawal, resting tachycardia, impaired blood pressure regulation, and orthostatic hypotension: these effects contribute to VVS in T2DM [146]. Recent data indicate that SGLT2i may mitigate autonomic dysfunction and reduce the recurrence of VVS, as shown in Table 1. In a prospective multicenter study of 324 T2DM individuals with VVS, SGLT2i use was associated with improved heart rate variability, better ^123I-metaiodobenzylguanidine (^123I-mIBG) indices, and a lower risk of VVS recurrence at 12 months [62]. These findings were reinforced by a large Chinese territory-wide cohort, where SGLT2i therapy was linked to a 51% reduction in new-onset syncope compared with DPP4i, with consistent benefits across most drug classes and patient subgroups [61]. Overall, while additional mechanistic studies are warranted, current evidence suggests that SGLT2i may reduce both the recurrence of VVS in T2DM and the incidence of new-onset syncope compared with alternative antidiabetic agents.

### Vascular effects

#### Amputation

Signals suggesting an increased risk of lower-limb amputation (LLA) emerged from early canagliflozin trials, but subsequent randomized studies and meta-analyses have not confirmed a consistent class-wide association. Advanced T2DM is strongly associated with micro- and macrovascular complications that predispose patients to LLA, with reported incidence rates between 1.5 and 5.0 per 1,000 patient-years. Common contributors include cardiovascular disease, peripheral neuropathy, peripheral arterial disease, and infection—all of which increase the likelihood of chronic ulcers and LLA [147, 148]. SGLT2i, via osmotic diuresis and intravascular volume contraction, were initially suspected to heighten ischemic risk and LLA [149, 150]. The CANVAS trial notably flagged an elevated risk of LLA with canagliflozin, yet subsequent RCTs and population data have not consistently replicated this finding: a comprehensive meta-analysis involving over 2 million patients confirmed no significant difference in LLA risk between SGLT2i and DPP-4i users, while suggesting a non-significant tendency toward higher risk compared to GLP-1 receptor antagonists, as shown in Table 1 [64]. Similarly, comparisons with DPP-4i or metformin do not show an increased risk [63, 64]. Furthermore, a subgroup analysis revealed that female sex (HR:2.78; 95%CI:1.12–6.94) and high-intensity statin use (HR:2.68; 95%CI:1.18–8.20) were associated with a higher LLA risk in SGLT2i users compared to metformin-treated patients [64]. Observational data indicate similar LLA incidence compared to placebo (HR:0.98; 95%CI:0.68–1.41) [64]. Overall, the preponderance of evidence indicates no consistent increase in amputation risk with SGLT2 inhibitors in T2DM, with no increased risk among SGLT2i and between SGLT2i and other anti-diabetic drugs [151]. However, high-risk subgroups - such as those with advanced peripheral arterial disease, previous ulcers or amputations, or multiple vascular comorbidities - warrant close monitoring and preventive measures during SGLT2i therapy.

### Metabolic effects

#### Lipid metabolism alterations

SGLT2i therapy is associated with modest and heterogeneous changes in lipid profiles, reflecting complex metabolic adaptations rather than clinically meaningful atherogenic risk that should be cautiously interpreted in the context of a global cardiovascular risk reduction strategy. Across large RCT datasets, SGLT2 inhibition is associated with small increases in LDL-C and HDL-C and a modest reduction in triglycerides, with effect sizes that are generally consistent across agents and study populations [30] In a meta-analysis of 60 RCTs, SGLT2i increased total cholesterol, LDL-C, and HDL-C while decreasing triglycerides, supporting that these changes represent a class effect rather than a molecule-specific signal [30]. Mechanistically, these lipid changes are thought to reflect metabolic reprogramming toward greater lipolysis and fatty-acid oxidation (with mild increases in ketone availability), alongside hemoconcentration and changes in hepatic lipid handling driven by negative energy balance [30]. From a clinical standpoint, the modest LDL-C rise should be interpreted in the context of consistent cardiovascular risk reduction observed in outcome trials, but a lipid profile check after initiation is reasonable in individuals at high baseline LDL-C or with recent lipid-therapy adjustments. 

#### Body weight reduction

Weight loss is a consistent pharmacodynamic consequence of SGLT2i therapy, largely attributable to urinary caloric loss from glycosuria, with additional contributions from natriuresis-related early fluid loss and longer-term fat mass reduction [31]. In a meta-analysis of 116 randomized controlled trials, SGLT2 inhibition reduced body weight by about 1.8 kg versus placebo, with broad consistency across follow-up durations, comorbidities, diabetes status, and SGLT drug types [31]. This magnitude is clinically relevant because even modest weight reduction can improve cardiometabolic risk factors and may support adherence by aligning treatment with patient-prioritized outcomes (weight and blood pressure). In routine practice, weight loss should prompt reassessment of concomitant glucose-lowering therapies (particularly insulin and sulfonylureas) to reduce hypoglycemia risk, while preserving the cardiorenal benefits that are largely independent of weight change.

#### NAFLD/MASLD reduction

Emerging evidence suggests that SGLT2i exert beneficial effects on metabolic liver diseases, likely mediated by weight reduction, improved insulin sensitivity, and altered hepatic substrate utilization. NAFLD/MASLD is a frequent comorbidity in T2DM and cardiometabolic disease, and SGLT2i have shown signal-level improvements in hepatic steatosis, as measured by imaging-based biomarkers, alongside reductions in liver enzymes in several trials [66]. In the E-LIFT RCT, empagliflozin improved liver fat content assessed by MRI-derived proton density fat fraction in individuals with T2DM and NAFLD, supporting a direct or indirect beneficial effect on ectopic hepatic fat [66]. A recent meta-analysis underlined that T2DM subjects with NAFLD/MASLD treated with SGLT2i had reduced liver function tests, a lower BMI and a better lipidic and glycemic profile [152]. An updated systematic review and meta-analysis of RCTs found that SGLT2i improved measures of steatosis and suggested modest improvements in fibrosis surrogates, though certainty was low-to-moderate and heterogeneity remained relevant [68]. Earlier systematic evidence in NAFLD with T2DM similarly reported improvements in steatosis and fibrosis surrogates with SGLT2i, reinforcing that liver-related benefits may track with improvements in weight, insulin resistance, and adipose tissue dysfunction rather than representing a licensed “liver-directed” indication [67]. Therefore, in individuals with T2DM and coexisting NAFLD/MASLD, SGLT2i can be considered a reasonable therapeutic choice when otherwise indicated, while clinicians should avoid presenting them as approved NAFLD/MASLD pharmacotherapy and should continue the standard management [68].

## Conclusion and clinical insights

SGLT2i have evolved from glucose-lowering agents to broad cardiometabolic and nephroprotective drugs with pleiotropic activity. Beyond their well-established cardiovascular and kidney-protective effects, accumulating evidence from RCTs, meta-analyses, and post-marketing surveillance indicates that several effects initially perceived as adverse or incidental, such as changes in renal hemodynamics, hematocrit, uric acid handling, and volume status, have emerged as mechanistically informative and, in selected populations, clinically advantageous.

The most consistent class-related signal remains an increased risk of GIs, which are generally manageable through counselling and timely topical antifungal therapy, and only rarely necessitate treatment discontinuation. The association with UTIs is weak in T2DM. Still, it emerges modestly among non-diabetic individuals with HF or CKD, underscoring the value of baseline risk assessment and patient education rather than routine drug cessation. SGLT2i consistently reduce AKI risk across diverse populations, consistent with their mechanistic benefits on tubuloglomerular feedback and intrarenal hemodynamics. EDKA risk, although uncommon, requires the adoption of standardised preventive measures, particularly in the perioperative setting and during episodes of acute illness. While accumulating data from urgent surgical contexts is reassuring, current recommendations continue to favour temporary withholding before elective procedures. Concerns regarding skeletal health and amputations have evolved: the early fracture risk signal with canagliflozin has not been confirmed by subsequent studies and meta-analyses. Overall, amputation risk does not appear elevated in SGLT2i compared with other antidiabetic drugs. Nonetheless, a potential relative disadvantage compared with GLP-1RA warrants attention when selecting the second-line antidiabetic drug in patients with severe PAD. Erythrocytosis is increasingly recognised as a treatment-related effect: although typically benign, it can occasionally unmask underlying clonal disorders, justifying a structured diagnostic assessment. From a clinical perspective, evidence supports maintaining SGLT2i therapy during mild to moderate GIs and prioritising careful adjustment of concomitant antihypertensive or diuretic regimens in patients at risk of hypotension before considering discontinuation. It is important to underline that the direction of certain effects, as hypotension and volume depletion, is strictly dependent on patient’s characteristics, comorbidities, and clinical context that determine whether these effects represent risk, neutrality, or potential benefit. Emerging data suggest favourable impacts on body weight, visceral adiposity, nephrolithiasis risk, and metabolic liver disease, effects that were not anticipated at the time of drug approval and remain underrecognized in routine practice. Accumulating evidence from RCTs, meta-analyses, and real-world data can help the clinician in balancing risks and benefits when prescribing SGLT2i, identifying individuals at higher risk for adverse events, and recognising clinical scenarios in which downstream metabolic and renal effects may be advantageous. As indications for SGLT2i continue to expand across patient populations, a nuanced understanding of their integrated safety and pleiotropic profile is essential to optimise individualised therapy.

## Supplementary Information

Below is the link to the electronic supplementary material.

Supplementary Material 1.

## Data Availability

No datasets were generated or analysed during the current study.

## Abbreviations

AE: Adverse Event
ADA: American Diabetes Association
AKI: Acute Kidney Injury
ALT: Alanine Aminotrasferase
ATP: Adenosine Triphosphate
BMI: Body Mass Index
BMD: Bone Mineral Density
BP: Blood Pressure
CANVAS: Canagliflozin Cardiovascular Assessment Study
CI: Confidence Interval
CKD: Chronic Kidney Disease
CV: Cardiovascular
DKA: Diabetic Ketoacidosis
DPP-4 / DPP-4i: Dipeptidyl Peptidase-4 (inhibitors)
EDKA: Euglycemic Diabetic Ketoacidosis
eGFR: Estimated Glomerular Filtration Rate
EMA: European Medicines Agency
EMPA-REG Outcome: Empagliflozin Cardiovascular Outcome Event Trial in Type 2 Diabetes Mellitus Patients
EPO: Erythropoietin
FDA: Food and Drug Administration
FG: Fournier’s Gangrene
GI, GIs: Genital Infection(s)
GLP-1: GLP-1RA Glucagon-Like Peptide-1 (Receptor Agonists)
HbA1c: Glycated hemoglobin
Hb: Hemoglobin
Hct: Hematocrit: HIF: Hypoxia inducible factor
HF: Heart Failure
HR: Hazard Ratio
KDIGO: Kidney Disease: Improving Global Outcomes
JAK2: Janus Kinase 2
LDL: Low-density lipoprotein
LLA: Lower limb amputation
MASLD: Metabolic dysfunction–associated steatotic liver disease
NAFLD: Non-alcoholic fatty liver disease
mIBG / ^123I-mIBG: Metaiodobenzylguanidine
Na⁺/K⁺-ATPase: Sodium–Potassium ATPase
OR: Odds Ratio
PAD: Peripheral Artery Disease
PY: Patient-Years
PV: Polycythemia Vera
RAAS: Renin–angiotensin–aldosterone system
RCT, RCTs: Randomized Controlled Trial(s)
RR: Risk Ratio / Relative Risk
SBP: Systolic Blood Pressure
SGLT1: Sodium-Glucose Cotransporter-1
SGLT2: Sodium-Glucose Cotransporter-2
SGLT2i: Sodium–Glucose Cotransporter-2 inhibitor
T2DM: Type 2 Diabetes Mellitus
TG: Triglycerides
UTI, UTIs: Urinary Tract Infection(s)
VVS: Vaso-Vagal Syndrome
WMD: Weighted Mean Difference
