# Supplementary material for: Beyond safety: adverse events and unanticipated advantages of SGLT2 inhibitors
Source: Eur J Clin Pharmacol. 2026 Mar 11;82(4):93. doi: 10.1007/s00228-026-04023-9 (PMC12979277; doi:10.1007/s00228-026-04023-9)
Supplement: Supplementary file 1 — Supplementary Material 1. [file 228_2026_4023_MOESM1_ESM.docx]

**Beyond Safety: Adverse Events and Unanticipated Advantages of SGLT2 Inhibitors**

Lorenzo Falsetti, Nicola Tarquinio, Luciano Mucci, Silvia Santini, Emanuele Guerrieri, Laura Giovenali, Giulia Pierdomenico, Vincenzo Zaccone, Giovanna Viticchi and Gianluca Moroncini

**SUPPLEMENTARY ONLINE ONLY MATERIAL**

**Urinary Tract Infections (48 results)**

*In type 2 diabetes mellitus (35 results):*

- Filters applied: in the last 10 years, English, Humans, Adult: 19+ years, MEDLINE.
- MeSH String: ("Sodium-Glucose Transporter 2 Inhibitors"[Mesh] AND "Urinary Tract Infections"[Mesh]) AND "Diabetes Mellitus, Type 2"[Mesh]

*In heart failure (7 results):*

- Filters applied: in the last 10 years, MEDLINE.
- MeSH String: ("Sodium-Glucose Transporter 2 Inhibitors"[Mesh] AND "Urinary Tract Infections"[Mesh]) AND "Heart Failure"[Mesh]

*In chronic kidney disease (8 results):*

- Filters applied: in the last 10 years, MEDLINE.
- MeSH String: ("Sodium-Glucose Transporter 2 Inhibitors"[Mesh] AND "Urinary Tract Infections"[Mesh]) AND "Renal Insufficiency, Chronic"[Mesh]

**Genital Infections (38 results)**

*In type 2 diabetes mellitus (29 results):*

- Filters applied: in the last 10 years, MEDLINE.
- MeSH String: ("Sodium-Glucose Transporter 2 Inhibitors"[Mesh] AND "Reproductive Tract Infections"[Mesh]) AND "Diabetes Mellitus, Type 2"[Mesh]

*In heart failure (4 results):*

- Filters applied: in the last 10 years, MEDLINE.
- MeSH String: ("Sodium-Glucose Transporter 2 Inhibitors"[Mesh] AND "Reproductive Tract Infections"[Mesh]) AND "Heart Failure"[Mesh]

*In chronic kidney disease (5 results):*

- Filters applied: in the last 10 years, MEDLINE.
- MeSH String: ("Sodium-Glucose Transporter 2 Inhibitors"[Mesh] AND "Reproductive Tract Infections"[Mesh]) AND "Renal Insufficiency, Chronic"[Mesh]

**Fournier Gangrene (14 results)**

- Filters applied: in the last 10 years, English, Humans, Adult: 19+ years, MEDLINE.
- MeSH String: "Sodium-Glucose Transporter 2 Inhibitors"[Mesh] AND "Fournier Gangrene"[Mesh]

**Acute Kidney Injury (55 results)**

- Filters applied: in the last 10 years, English, Humans, Adult: 19+ years, MEDLINE.
- MeSH String: ("Sodium-Glucose Transporter 2 Inhibitors"[Mesh]) AND "Acute Kidney Injury"[Mesh]

**Nephrolitiasis (6 results)**

- Filters applied: in the last 10 years, MEDLINE.
- MeSH String: ("Sodium-Glucose Transporter 2 Inhibitors"[Mesh]) AND "Kidney Calculi"[Mesh]

**Polycytemia (26 results)**

- Filters applied: in the last 10 years, MEDLINE.
- MeSH String: ("Sodium-Glucose Transporter 2 Inhibitors"[Mesh]) AND "Polycythemia"[Mesh]

**Euglycaemic ketoacidosis (178 results)**

- Filters applied: in the last 10 years, English, Humans, Adult: 19+ years, MEDLINE.
- MeSH String: ("Sodium-Glucose Transporter 2 Inhibitors"[Mesh] AND "Diabetic Ketoacidosis"[Mesh]

**Fractures and osteoporosis (80 results)**

*Fractures (59 results):*

- Filters applied: in the last 10 years, MEDLINE.
- MeSH String: ("Sodium-Glucose Transporter 2 Inhibitors"[Mesh] AND "Fractures, Bone"[Mesh]

*Osteoporosis (3 results):*

- Filters applied: in the last 10 years, MEDLINE.
- MeSH String: ("Sodium-Glucose Transporter 2 Inhibitors"[Mesh] AND "Osteoporosis"[Mesh]

*Bone mineral density (18 results)*

- Filters applied: in the last 10 years, MEDLINE.
- MeSH String: ("Sodium-Glucose Transporter 2 Inhibitors"[Mesh] AND "Bone Density"[Mesh]

**Hypotension and dehydration (27 results)**

*Hypotension (14 results):*

- Filters applied: in the last 10 years, MEDLINE.
- MeSH String: ("Sodium-Glucose Transporter 2 Inhibitors"[Mesh] AND "Hypotension"[Mesh]

*Dehydration (13 results):*

- Filters applied: in the last 10 years, MEDLINE.
- MeSH String: ("Sodium-Glucose Transporter 2 Inhibitors"[Mesh] AND "Dehydration"[Mesh]

**Syncope (3 results)**

- Filters applied: in the last 10 years, MEDLINE.
- MeSH String: ("Sodium-Glucose Transporter 2 Inhibitors"[Mesh] AND "Syncope"[Mesh]

**Amputations (67 results)**

- Filters applied: in the last 10 years, MEDLINE.
- MeSH String: ("Sodium-Glucose Transporter 2 Inhibitors"[Mesh] AND "Amputation, Surgical"[Mesh]
